# Challenges in the measurement and interpretation of dynamic functional connectivity

**DOI:** 10.1162/imag_a_00366

**Published:** 2024-11-19

**Authors:** Timothy O. Laumann, Abraham Z. Snyder, Caterina Gratton

**Affiliations:** Department of Psychiatry, Washington University in St. Louis, St. Louis, MO, United States; Department of Radiology, Washington University in St. Louis, St. Louis, MO, United States; Department of Neurology, Washington University in St. Louis, St. Louis, MO, United States; Department of Psychology, Florida State University, Tallahassee, FL, United States; Department of Neuroscience, Florida State University, Tallahassee, FL, United States; Department of Psychology, University of Illinois at Urbana-Champaign, Urbana-Champaign, IL, United States; Beckman Institute, University of Illinois at Urbana-Champaign, Urbana-Champaign, IL, United States

**Keywords:** dynamic functional connectivity, resting-state fMRI, BOLD fMRI dynamics, spontaneous activity, sampling variability, stationarity

## Abstract

In functional MRI (fMRI), dynamic functional connectivity (dFC) typically refers to fluctuations in measured functional connectivity on a time scale of seconds. This perspective piece focuses on challenges in the measurement and interpretation of functional connectivity dynamics. Sampling error, physiological artifacts, arousal level, and task state all contribute to variability in observed functional connectivity. In our view, the central challenge in the interpretation of functional connectivity dynamics is distinguishing between these sources of variability. We believe that applications of functional connectivity dynamics to track spontaneous cognition or as a biomarker of neuropsychiatric conditions must contend with these statistical issues as well as interpretative complications. In this perspective, we include a systematic survey of the recent literature, in which sliding window analysis remains the dominant methodology (79%). We identify limitations with this approach and discuss strategies for improving the analysis and interpretation of sliding window dFC by considering the time scale of measurement and appropriate experimental controls. We also highlight avenues of investigation that could help the field to move forward.

## 1 Introduction

BOLD fMRI has been used to map the representation of function within the brain since the early 1990s (Bandettini et al., 1992;Kwong et al., 1992). Remarkably, even in the absence of imposed cognitive manipulations, spontaneous BOLD signals exhibit correlated fluctuations that reflect the architecture of known functional systems or networks. This phenomenon is known as resting-state functional connectivity (FC) after the unconstrained state in which it was first observed (Biswal et al 1995). Since its discovery, many researchers have used resting-state FC to examine “static” features of functional network organization that are assumed to be invariant over the period of the recording, both in healthy subjects and in populations with various neuropsychiatric disorders (Bruin et al., 2023;Greicius, 2008;Javaheripour et al., 2021;Sheffield & Barch, 2016).

Importantly, functional connectivity changes over many time scales: over years with development (Graham et al., 2015), over hours in relation to circadian rhythms and sleep (Shannon et al., 2013;Tagliazucchi & Laufs, 2014), and over minutes as participants engage in various cognitive tasks (Cole et al., 2014;Krienen et al., 2014). FC has also been observed to vary over short time scales (i.e., less than a minute) in the task-free state (Allen et al., 2014;Chang & Glover, 2010;Hutchison et al., 2013b). Following these studies, we use “dFC” to refer to strategies that aim to estimate change in fMRI functional connectivity over short time scales, with a particular focus on “sliding windows” dFC (seeBox 1). There are many alternative strategies to study dynamics in fMRI time series covered in prior reviews (Hutchison et al., 2013a;Keilholz et al., 2017;Liegeois et al., 2017,^2021^;Lurie et al., 2020;Pervaiz et al., 2022;Preti et al., 2017). Our objective is not to recapitulate this material. Rather, we aim to emphasize and clarify statistical and interpretive issues that arise in fMRI dFC analysis as it is most frequently employed in the current literature. These issues have been raised in prior reports, though not with the present emphasis (Hindriks et al., 2016;Laumann et al., 2017;Liegeois et al., 2017;Lurie et al., 2020;Preti et al., 2017).

Box 1. Stationary versus non-stationary FC

A process is said to be**stationary**if the expected values of statistics that characterize that process are constant over time (Liegeois et al., 2017;Lurie et al., 2020). Importantly, stationarity does not imply that measured values are invariant over time. For example, the position of a frictionless pendulum changes as a function of time. However, provided no external force is applied, the period of the pendulum (i.e., how long it takes to complete one full swing) is stationary. Special importance attaches to the observation of non-stationarity in a brain measure because it implies change in an underlying physiological process. For example, changes in the EEG occipital alpha rhythm between resting wakefulness, drowsiness, and REM sleep suggest that different neural assemblies underlie these distinct brain states (Cantero et al., 2002). Notably, non-stationary EEG features reflect changes in electrical activity on a time scale of hundreds of milliseconds, which is very different from the time scale of fMRI-based FC (seeSection 2.1;Box 2).

**“Functional connectivity” (FC)**refers to measurement of the extent to which fMRI signal pairs are correlated. Conventional FC implicitly assumes stationarity, that is, that BOLD signal correlations are stationary over the period of measurement (typically, 5–10 min). Methods for evaluating static FC include linear correlation (Biswal et al., 1995;Fox et al., 2005) and static independent component analysis (Beckmann et al., 2005).

Sliding window correlation and its variants constitute the most commonly used method to assess fMRI dynamics at short time scales (Fig. 2). Classic**sliding window correlation**is computed by evaluating FC over short epochs (windows), typically 30–120 sec, followed by K-means clustering (Allen et al., 2014). This procedure leads to a description of fMRI data as a sequence of distinct FC matrices which are then clustered into states. The underlying assumption in sliding window correlation is that FC change over these windows reflects a change in brain state (rather than FC sampling error). Thus, sliding window correlation explicitly aims to identify non-stationarity. Returning to the pendulum analogy, this is comparable with an external force changing the speed or direction of the swing. Analysis of change in functional connectivity amounts to evaluation of the variance of a second-order statistic (FC), and is, therefore, a fourth-order statistic (seeBox 2). Other sliding window-based methods assess dynamics by computing the variance of derived measures of FC. Such other derived measures might include the product of fMRI**temporal derivatives**(Mai et al., 2022),**Granger causality estimates**(Qin et al., 2022), and**graph-theoretic measures**such as node degree, clustering coefficient, etc. (Li et al., 2022) (and more; this is not an exhaustive list). We refer to all such window-based methods that seek to identify non-stationarity in functional connectivity as “dFC,” and they are the focus of our article.

A second class of methods for studying BOLD fMRI dynamics aims to identify transient FC events, that is, instantaneous coactivation patterns in BOLD signals. Examples include**point-process analysis**(Tagliazucchi et al., 2012),**hemodynamic deconvolution**of events (Petridou et al., 2013),**coactivation patterns**(CAPs;Liu & Duyn, 2013), and**instantaneous co-activation patterns**(iCAPs;Karahanoglu & Van De Ville, 2015). These approaches identify extreme excursions of the fMRI signal at the periphery of state space. These events are first order in fMRI signal (Box 2). A related method focuses on**extreme excursion events**of pairwise fMRI signal relations, which are second order (Sporns et al., 2021;Zamani Esfahlani et al., 2020). Notably, the existence of extreme excursions in state space is formally agnostic with regard to stationarity versus non-stationarity. By analogy, the swinging pendulum reaches extreme excursions twice in every cycle. The kinetics of the pendulum (including its extremes) are fully described by a stationary sine wave. Importantly, whether an extreme excursion of fMRI time series represents a non-stationary feature can be adjudicated by reference to a stationary null model of the fMRI signal, which specifies, for example, its covariance structure (seeLadwig et al., 2022;Laumann et al., 2017;Liegeois et al., 2017).

Many other methods that do not rely on sliding windows have been proposed to capture dynamic features of fMRI data. For example, whereas FC conventionally is evaluated at zero lag, it is also possible to measure FC at**non-zero lag**(Mitra et al., 2014). FC lag structure is informative regarding the propagation of BOLD signals between brain regions (Bolt et al., 2022). Signal propagation can also be studied using**autoregressive modeling**(Liegeois et al., 2019). In the context of the pendulum analogy, these models are akin to one pendulum swinging into another pendulum with the same period (again, stationary in the absence of external forces). Other methods to evaluate fMRI dynamics include**Leading Eigenvector Dynamics Analysis**(Cabral et al., 2017;Farinha et al., 2022) and**Hidden Markov Models**(Shappell et al., 2019;Vidaurre et al., 2017), which model brain activity as a succession of states. Other generative models have been used to describe dynamic features of fMRI data including the**General Laplacian Mixture Model**(GLMM;Pervaiz et al., 2022) and**Multi-dynamic Adversarial Generator Encoder**(MAGE) modeling (Ricchi et al., 2022). Crucially, all the latter approaches do not explicitly assume non-stationarity (seeLiegeois et al. (2017)for expanded discussion). However, it is possible to use these methods to demonstrate non-stationarity through comparison with a suitable stationary null model (as in, e.g.,Ricchi et al., 2022). In our perspective, we do not discuss these alternative methods in detail.

**Fig. 1.**
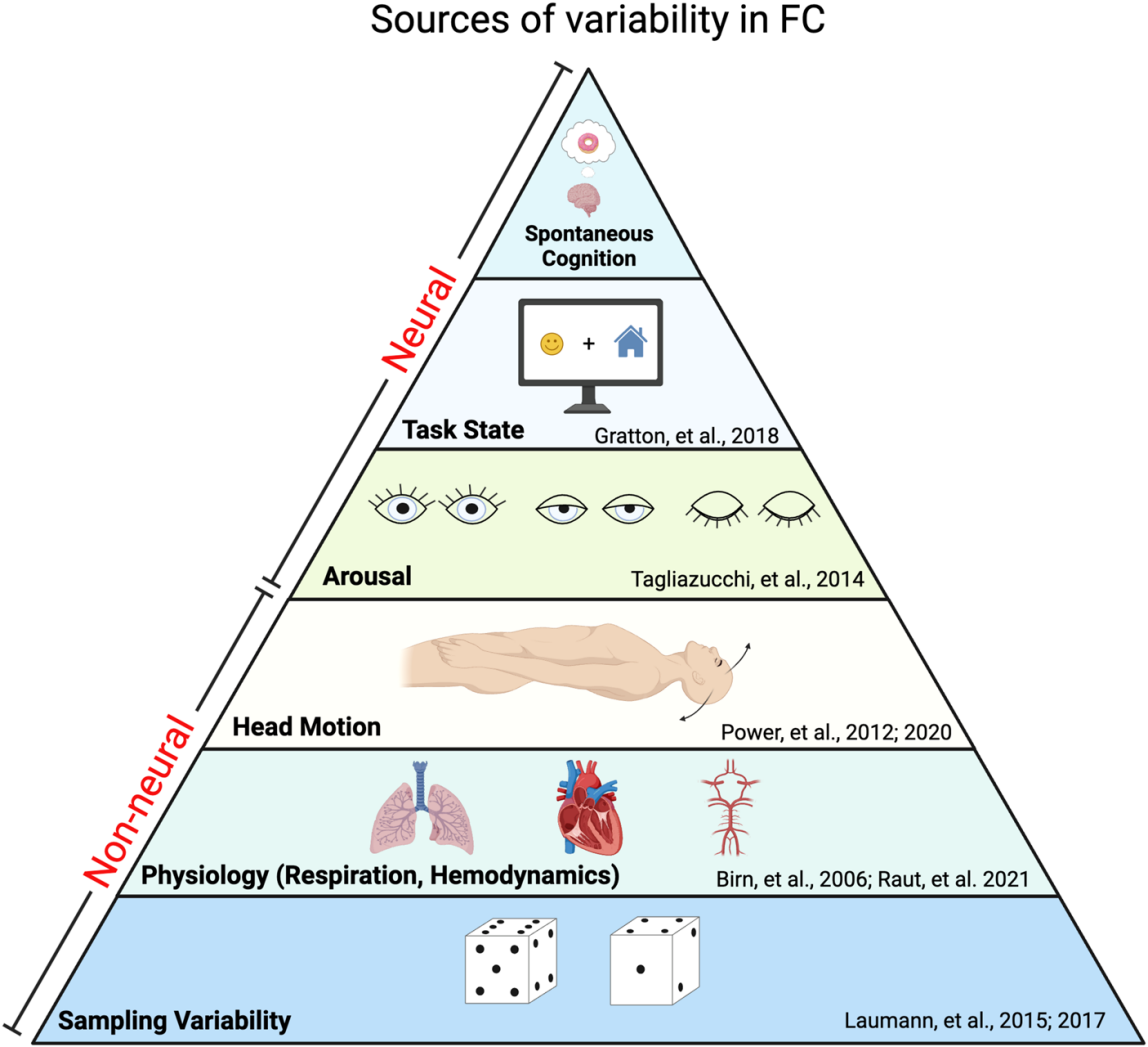
Non-neural and neural factors contribute to variability in functional connectivity. This figure depicts sources of variability in fMRI FC, approximately ordered according to the magnitude of their contribution to functional connectivity variance. Non-neural sources of variability include sampling variability and physiological biases/artifacts. The relative order of these factors may depend on the duration of the sample on which a given FC measure is derived. In particular, sampling variability is inversely proportional to duration. (Created in BioRender. Laumann, T. (2023)BioRender.com/i95n037).

Much of the interest in dFC is inspired by the potential to track fluctuating cognition (Gonzalez-Castillo et al., 2015;Kucyi, 2018;Shine & Poldrack, 2018), and as a source of biomarkers of neuropsychiatric conditions (Filippi et al., 2019). A natural intuition underlying these research objectives is that relevant neural dynamics can be measured using fMRI FC, much as has been done with EEG and task-based fMRI. However, variability in fMRI FC of the order of seconds can arise from several distinct mechanisms (Fig. 1) including sampling error, artifacts generated by head motion or fluctuating arterial pCO_2_, and neurally driven changes associated with drowsiness and explicit task performance. Our perspective is that these potential sources of variability lead to challenges in the measurement and interpretation of fMRI dFC. In this context, we clarify critical concepts that apply to studies of FC dynamics:Box 1discusses stationarity versus non-stationarity in relation to analytic approaches.Box 2defines statistical order and time scale in relation to measures of brain mechanisms.

Box 2. Key properties of BOLD signals that impact interpretation of dFC

**Order of statistic:**Order refers to the power (i.e., the raised exponent) with which a measure enters into a computed statistic. The concept of order is related to moments of a distribution (e.g., mean = 1st order; variance = 2nd order; skewness = 3rd order; kurtosis = 4th order).

**Time scale:**Time scale refers to the span of time over which a phenomenon occurs. In characterization of physiological data, time scale is reflected in spectral content, that is, relative contribution of fast or slow frequencies. Ideally, there should be correspondence in the time scale between the phenomenon of interest and the measure of the phenomenon (seeSection 4.1for further discussion).

**Fig. 2.**
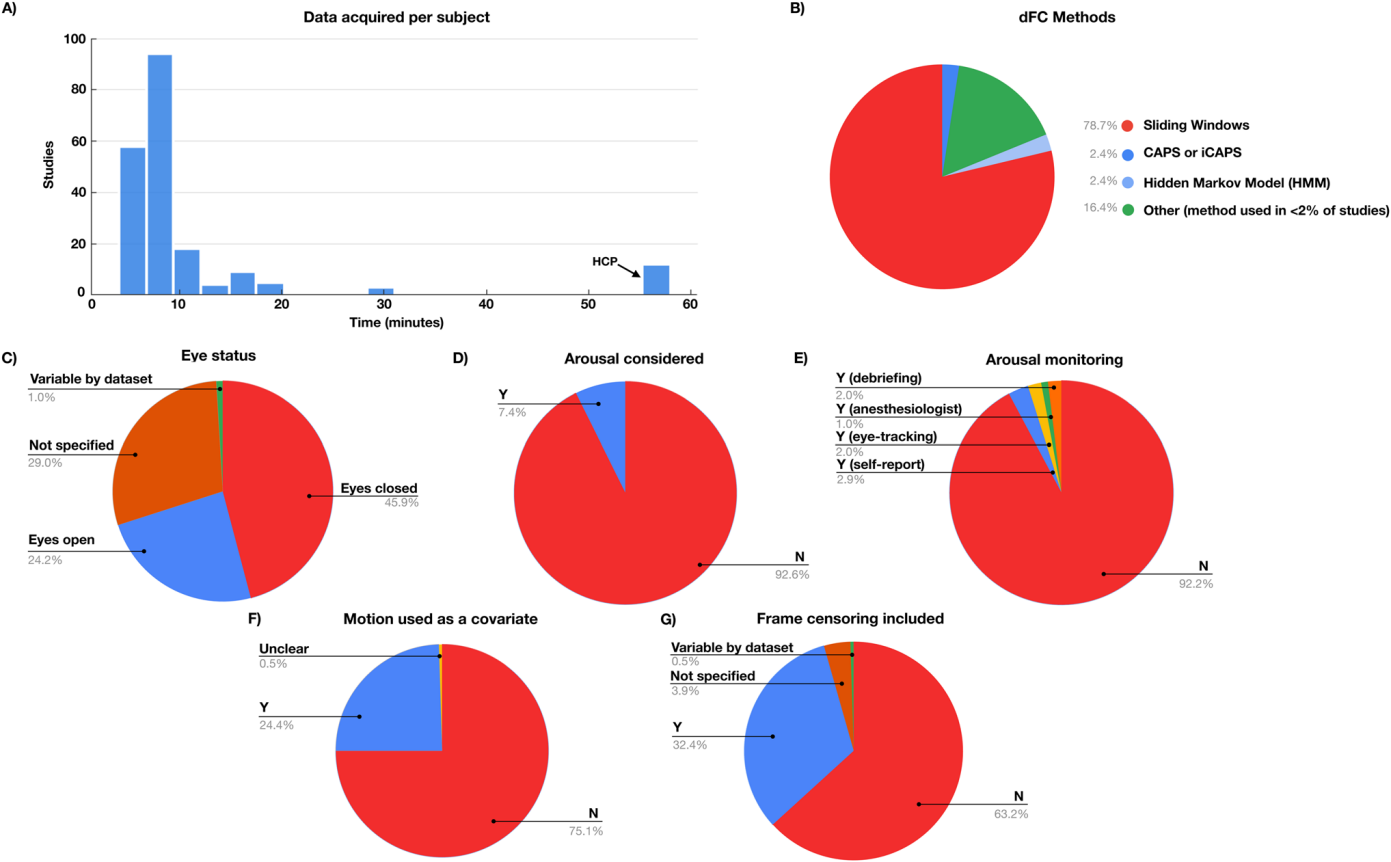
Summary of results from a survey of dynamic FC studies published in 2022 and the beginning of 2023 (Supplementary Table 1). (A) Distribution across dynamic FC studies of data acquired per subject. If multiple conditions/datasets were included in a single study, these were included as separate estimates in this histogram. The only studies that had greater than 30 min of data per participant were studies that used Human Connectome Project data. (B) Percent of studies using different methods for dynamic FC analysis (sliding window, HMM, etc.). Methods that were used in <2% of studies were grouped together in “Other”. (C) Percent of studies with eyes open versus eyes closed resting state. Eyes closed resting state is associated with higher likelihood of sleep (Tagliazucchi & Laufs, 2014). (D) Percent of studies that considered arousal as a potential cause of dFC. (E) Percent of studies where arousal was monitored during scans (and how arousal was monitored). (F) Percent of studies where motion was included as a covariate in statistical analysis. (G) Percent of studies where frame censoring of small movements was included in analysis.

As the field of dFC rapidly expands, the above-mentioned issues continue to impact a majority of publications. A PubMed query (April 18, 2024) with the search term “dynamic functional connectivity” identified more than 1,000 papers, 34 of which are reviews. To assess the recent state of the field, we include a limited survey of “dynamic functional connectivity” articles published in 2022 and the first half of 2023 (204 articles; see Supplementary Methods;Supplementary Table 1). Although many methods are reflected in our survey of the recent literature, we found that the vast majority of studies (78.7%) report variants of a “sliding window correlation” approach (Box 1). Accordingly, this is the focus of our discussion. Other methods, including Hidden Markov Models (2.4%) and co-activation patterns (2.4%), represent a much smaller portion of the literature (Fig. 2). Some of the principles discussed in this work may also extend to these other approaches to studying fMRI dynamics; however, although these methods assess fMRI dynamics, they do not necessarily evaluate non-stationarity (seeBox 1).

Sections2and3of this work discuss these concepts and their impact on sources of FC variability, first in relation to sampling error and physiological artifacts and then in relation to neural sources of variance.Section 4reviews applications of dFC in relation to cognition and biomarkers.Section 5discusses approaches to improve future studies of fMRI dynamics.

## 2 Non-Neural Sources of Variation in FC

### 2.1 Sampling variability largely accounts for apparent dFC

Sampling variability is a particularly important—but often under-recognized—issue in measures of FC (Fig. 1). Sampling variability (or “sampling error”) is inversely proportional to the number of independent measurements. All measures are subject to sampling error. However, owing to the slow temporal scale of BOLD fMRI signals, sampling error is especially problematic in dFC, which relies on short observation periods. BOLD signals reflect neural activity on a time scale (Box 2) much slower than frequencies typically encountered in electrophysiology (Leopold & Maier, 2012), largely determined by the kinetics of neurovascular coupling (Hillman, 2014). In particular, neurally driven BOLD signals are primarily restricted to infra-slow frequencies (<0.2 Hz; although note that these may differ across brain areas^*^) (Anderson, 2008;Hathout et al., 1999) (but seeLewis et al., 2016). Thus, sliding window dFC must contend with accurately measuring correlations among signals fluctuating at <0.2 Hz with samples of 30–120 sec.

Sampling variability also depends on the**statistical order**of a measure (seeFig. 4). Order refers to the power (i.e., the raised exponent) with which a measure enters into a computed statistic (Box 2). The mean is a first-order statistic, whereas variance is a second-order statistic. Thus, the order of a statistic reflects increasingly complex aspects of the distribution of a measure. In the context of fMRI, task-evoked responses are first order because the response at each voxel enters into the measure with power one, usually averaged over trials. FC statistics are second order in fMRI signal because correlation is computed as the normalized product of two signals extracted from pairs of brain regions. As sliding window dFC estimates variance in FC, it is a fourth-order statistic. These order and temporal scale differences lead FC (and sliding window dFC) measures to fundamentally differ in their statistical properties from task-evoked fMRI measures.

**Fig. 3.**
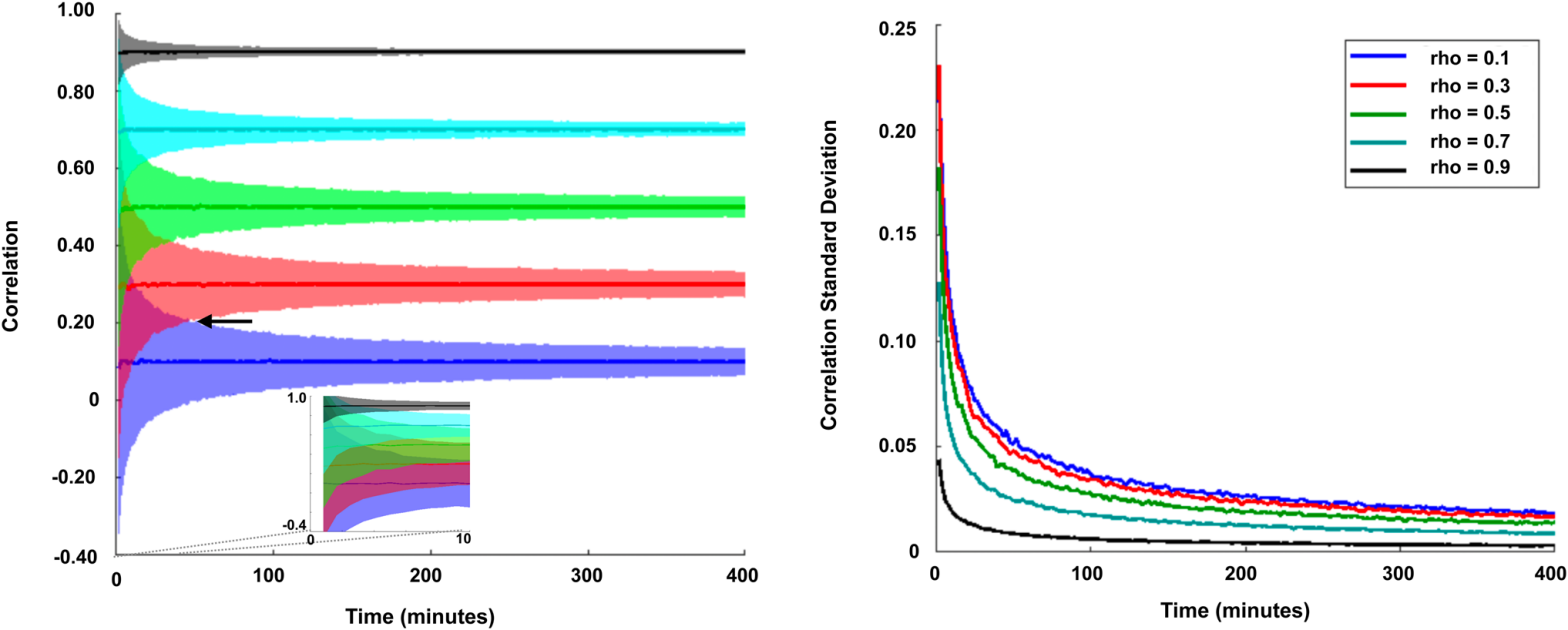
The effect of sampling error on measurement of static FC. Bivariate BOLD time series were simulated over a range of fixed correlation values (rho) over 1,000 realizations. The left plot illustrates measured correlation confidence intervals (+/- 1.96 SD) parametric in quantity of data. Note the expectation value of correlation matches rho with increasing precision as quantity of data increases. The confidence interval narrows as the quantity of data increases. Inset zooms in on confidence intervals for less than 10 min of data. Small quantities of data (<2 min., as is typical of sliding windows) do not permit distinguishing rho = 0.1 versus rho = 0.7, as the confidence intervals overlap; indeed, the arrow indicates where confidence intervals for rho = 0.1 and rho = 0.3 no longer overlap, which occurs at approximately 50 min. The right plot shows the standard deviation of correlation over 1,000 realizations parametric in quantity of data. Note that the standard deviation is least when correlations are strong (rho = 0.9) and greatest when data quantity is low (e.g., <2 min). N.B.: The power spectrum of the simulations matched real BOLD fMRI data reported inLaumann et al. (2015); thus, correlation estimate standard deviations at a given sample duration reflect degrees of freedom resulting from that particular processing pipeline. The intrinsic time scale of neural activity modestly varies across the brain (Raut et al., 2020); thus, correlation estimate standard deviations could also depend modestly on ROI selection.

Consider first the case of static FC. The dependence of FC sampling variability on data quantity has been empirically demonstrated using very large datasets acquired in individuals (Anderson et al., 2011;Gordon et al., 2017;Laumann et al., 2015). These studies show that reliable estimation of static FC requires large amounts of data (~30–100 min or more), depending on the spatial precision of the FC estimate and the extent to which BOLD signals are corrupted by artifact (Gordon et al., 2017;Laumann et al., 2015;Noble et al., 2019). Given the small amounts of data per individual typically obtained in resting-state studies (e.g., 5–10 min), sampling error in static FC is likely a major contributor to difficulty using FC as a biomarker. To illustrate this issue, we conducted a simulation of realistic BOLD fMRI data (Fig. 3). The confidence interval of FC derived from this simulation is inversely proportional to both the magnitude of the correlation and the quantity of available data. Uncertainty in the correlation estimate is greatest when correlations are weak and data quantity is small (compare shaded dark blue area with shaded black area in the left panel ofFig. 3). Thus, the ability to measure FC (or change in FC) over very short time windows (e.g., <2 min) is limited, especially if the correlation is weak (seeFig. 3, left panel inset). It should be noted this simulation reflects bivariate correlation; alternative strategies, for example, dimensionality reduction or data aggregation may reduce sampling variability. However, the basic principle that sampling error is inversely proportional to data quantity remains the same. In practice, we have found that identifying reliable dFC statistics can remain elusive even with multivariate strategies that include dimensionality reduction (Laumann et al., 2017).

**Fig. 4.**
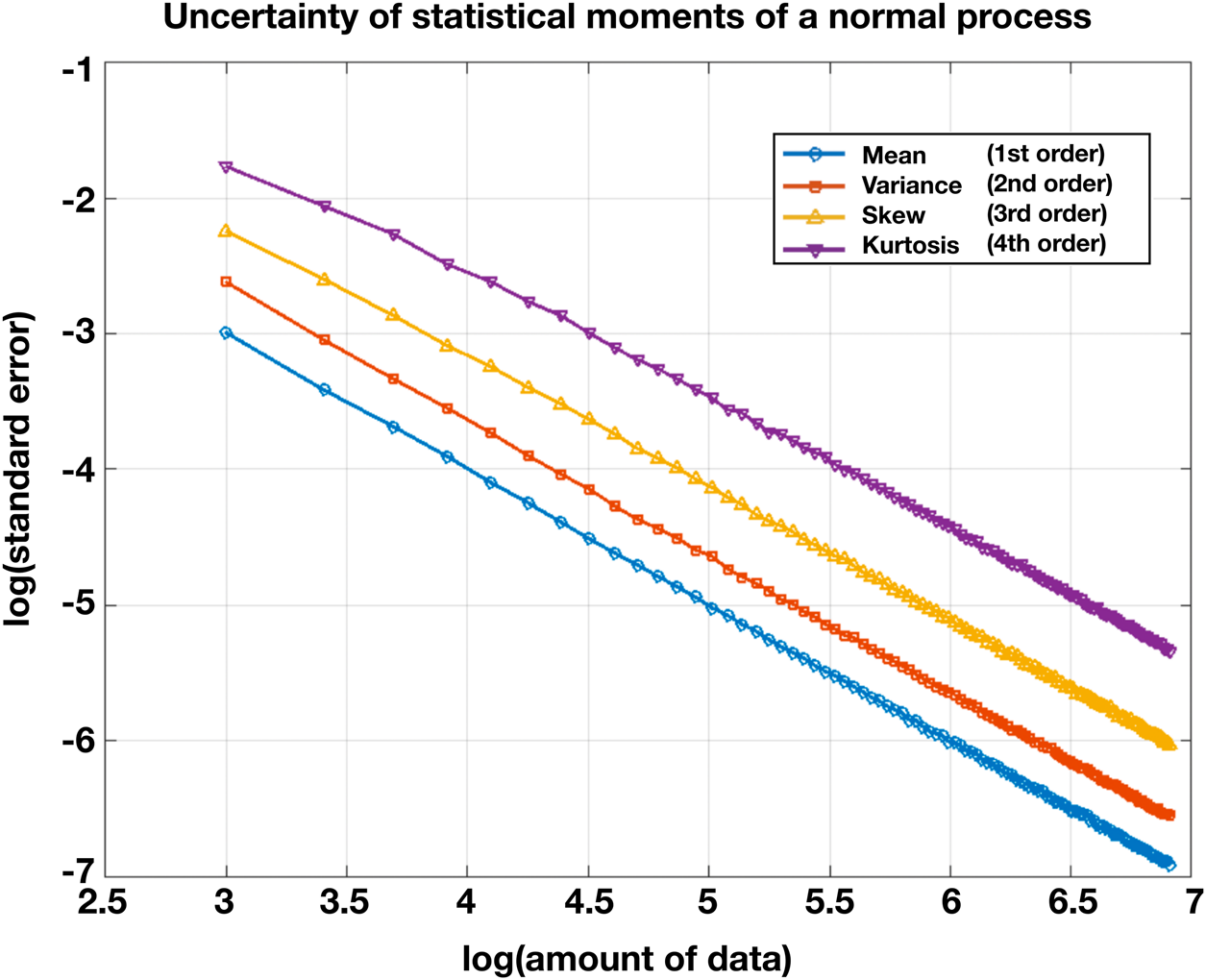
Uncertainty of statistics (moments of order 1, 2, 3, 4) derived from a random, normally distributed variate as a function of amount of data. Statistics were generated from increasing sample sizes of mean 1, standard deviation 1, random normal deviates. Ten thousand realizations were computed at each sample size. The y-axis depicts the log of the standard error of each estimate across realizations. Uncertainty of estimate increases with order (mean = 1st order; variance = 2nd order; skew = 3rd order; kurtosis = 4th order). Thus, increasingly large sample sizes are required to obtain equivalent reliability for higher order statistics.

Following this logic, the uncertainty imposed by sampling error is exacerbated in sliding window dFC. As FC is a second-order statistic (Box 2), dFC—which seeks to measure temporal variance in FC—represents a fourth-order statistic (a basic result in mathematic statistics holds that the variance of a statistic of order n is a statistic of order 2n;Weatherburn, 1962). The larger the order of a statistic, the larger the sampling error (Fig. 4). Notably, these considerations apply most directly to estimates of variance in FC (as in sliding windows). Measures of FC explicitly incorporating temporal lag (rather than non-stationarity), as used in, for example,Liegeois et al. (2021)andMitra et al. (2023), are second order in fMRI signal (as is static FC). Other measures of fMRI dynamics, for example, Hidden Markov Models (HMM), have other sampling error characteristics not discussed here.

Empirically, as expected fromFigure 4, sliding window dFC is less reliable than static FC. Publications that have evaluated sliding window dFC reliability generally report low intra-class correlation (ICC) values, that is, ICC < 0.5, depending on measurement details (Choe et al., 2017;Smith et al., 2018;Zhang et al., 2018,^2022^). In practice, reliable sliding window dFC measurements likely require sample durations of at least 10 min (Hindriks et al., 2016). Further, as noted for static FC above, more data may be required depending on the magnitude of the BOLD signal, spatial precision, and contrast-to-noise ratio of the fMRI pulse sequence (Triantafyllou et al., 2005). Parcellation scheme affects reliability depending both on how well the parcels match the underlying functional organization (e.g., group average templates mislocalize functional data in different individuals; (Gordon et al., 2017)) and the size of the parcels, which affects the area over which data are averaged. Reliability will be greatest for ROI pairs with strong correlation (Fig. 3). Thus, reliability of sliding window dFC tends to be greatest for ROI pairs that exhibit the greatest reliability of static FC. Importantly, as a general rule, increased window duration (i.e., data quantity) will improve reliability of an FC estimate (as illustrated inFig. 3).

We believe that the preceding considerations highlight the practical challenge of measuring FC dynamics and suggest the need for extended data acquisitions to achieve adequate statistical power. In the present survey of articles on FC dynamics, a median of 8 min. of data per participant was collected (seeFig. 2). The only papers approaching a full hour of data used one common dataset (the Human Connectome Project (HCP;Smith et al., 2013). Thus, most extant resting-state studies have not acquired enough data to reliably estimate static (zero-lag) FC within an individual, let alone more complex temporal structure.

With smaller amounts of data, sampling variability in FC estimates will be high, increasing apparent non-stationarity in FC estimates. To determine whether apparently dynamic FC represents true non-stationarity in FC as opposed to sampling error, it is useful to contrast results with a null model (Liegeois et al., 2021). For strong inference, the null model should accurately match the relevant static FC properties of the measured data: that is, the model and actual data should have the same static correlation structure, spectral content, and AR properties (seeLiegeois et al. (2017)for a more extensive discussion and specific strategies).

Consistent with our preceding discussion of sampling variability, measures of resting-state fMRI dynamics typically exhibit limited differences relative to null models (Casorso et al., 2019;Laumann et al., 2017;Liegeois et al., 2021). At least one recent paper suggests that the additional yield of dynamic FC estimates in comparison with static FC is slight (Matkovic et al., 2023). This is also true of single time point “event” analyses for which it has been shown that the likelihood of observing events between a pair of regions increases with the magnitude of static FC between them (Ladwig et al., 2022;Novelli & Razi, 2022). An explicit algebraic expression for this dependence is given inNadarajah and Pogány (2016). Thus, the presence of events or CAPs may be consistent with a static connectome. Stationary null models that include features related to signal propagation or lag (e.g., AR models;Casorso et al., 2019;Liegeois et al., 2017,^2019^) provide a more complete account of real BOLD fMRI data in comparison with purely zero-lag FC models. However, the presence of lag structure does not imply non-stationarity in FC (Box 1). Future work should expand the use of appropriate null models to determine whether dFC measures provide statistically significant evidence of non-stationarity. The value of such work would be enhanced by extended fMRI data collection in which small deviations from stationarity can be more reliably detected.

### 2.2 Variability from physiological biases and artifacts

In addition to sampling variability, FC measures are sensitive to fMRI artifacts and physiological biases from (even sub-millimeter) head motion and changes in arterial pCO_2_(Laumann et al., 2017;Power et al., 2012,2020a,2020b;Satterthwaite et al., 2012;Van Dijk et al., 2012). Such artifacts are widely distributed and systematically bias FC measurements throughout the brain (Laumann et al., 2017;Power et al., 2012,2020a,2020b;Satterthwaite et al., 2012;Van Dijk et al., 2012). Importantly, these influences can vary over short time scales with head motion or changes in respiration (Power et al., 2020a,2020b). Thus, non-neural signals and fMRI artifacts can lead to apparent dynamic changes in FC (Laumann et al., 2017).

The problem of artifact in the search for dFC biomarkers is exacerbated by the fact that head motion and respiration differ systematically across populations. Head motion is greater in many psychiatric samples relative to typical controls (Castellanos & Aoki, 2016;Picci et al., 2016), and greater in children than adults (Power et al., 2012). Head motion and respiration can also vary systematically across tasks (Birn et al., 2009;Engelhardt et al., 2017). Further, head motion is correlated with many behavioral measures (Siegel et al., 2017) and physical characteristics (Bolton et al., 2020).

Addressing these sources of artifacts and biases requires artifact reduction strategies, that is, “denoising” (e.g., strategies to reduce global respiratory signals and transient motion artifacts) (Power et al., 2015;Satterthwaite et al., 2019). The efficacy of such strategies has been extensively demonstrated in the context of static FC measurement (Ciric et al., 2017;Parkes et al., 2018;Power et al., 2014). Most dFC studies also implement some form of denoising (e.g., nuisance regression and/or outlier exclusion). However, many studies do so only to a limited extent. Only a minority of publications in our survey of the recent literature included some form of motion censoring (32%), which, when combined with global signal attenuation (e.g., via global signal regression or CompCor), represents the best performing strategy for reducing motion biases in static FC (Ciric et al., 2017). Additional strategies have been proposed for addressing motion at later stages of analysis, such as using motion-related measures as covariates (Satterthwaite et al., 2019). However, these strategies are still relatively infrequently employed (24%).

Denoising has been shown to be especially challenging in the context of sliding window dFC in which transient effects have outsized impact (Nalci et al., 2019). Thus, we believe an important future avenue of research is the development and systematic evaluation of strategies that address the specific challenge of head motion and physiological artifact denoising in dFC. Of note,Joliot et al. (2024)have recently reported that global signal regression was the most effective method for removing FC differences between drowsy and alert states, which may contribute to observations of dFC for both neural and non-neural (e.g., associated respiratory changes, seeSection 3.2) reasons. They additionally propose Gaussian standardization of the FC distribution as a less aggressive approach to minimizing arousal effects.

## 3 Neural Sources of Rapid Variation in FC

Given the considerations raised inSection 2, it is useful to review characteristics of well-documented non-stationarity in FC. In the following section, we consider neurally driven changes in FC during experimentally imposed tasks and with alterations in arousal.

### 3.1 Task states are associated with modest levels of variation in FC

FC is altered when participants perform tasks across a wide range of cognitive domains, from sensory motor learning paradigms (Bassett et al., 2011) to working memory (Cohen et al., 2014;Finc et al., 2020;Shine et al., 2016), sustained attention (Al-Aidroos et al., 2012;Rosenberg et al., 2016), and movie watching (Betti et al., 2013). Task-induced changes can be relatively widespread, occurring within and between different networks in the cortex and sub-cortex (Betti et al., 2013;Gratton et al., 2016;Krienen et al., 2014). Some FC changes may represent “task-general” effects (Cole et al., 2014;Gratton et al., 2016;Krienen et al., 2014), while others are specific to particular cognitive demands (Al-Aidroos et al., 2012;Cole et al., 2014;Gratton et al., 2016;Krienen et al., 2014;Porter et al., 2023;Rosenberg et al., 2016;Shirer et al., 2012). Importantly, unbiased estimation of task-based changes in FC requires removal of the task-evoked signal to avoid conflation of FC estimates with evoked signals (Cole et al., 2019). These task-related effects are sufficiently consistent such that it is possible to decode task state (using a previously trained classifier) on the basis of FC patterns (Gonzalez-Castillo et al., 2015;Porter et al., 2023;Rosenberg et al., 2016;Shirer et al., 2012).

A number of physiological mechanisms have been proposed to underlie changes in FC in different task contexts. Changes in FC could arise because neural signals in task-relevant brain regions become more coherent (Nir et al., 2007). This idea is linked to theories of “communication through coherence” (CTC), derived from related observations of local field potentials in macaques (Fries, 2005). Other evidence suggests that tasks suppress spontaneous activity in engaged areas, a phenomenon referred to as “stimulus quenching” (Churchland et al., 2010;Cohen & Maunsell, 2009;He, 2013;Ito et al., 2020). Selective suppression of spontaneous activity could influence BOLD FC between regions. Changes in FC with task states (and arousal) may also be mediated by changes in the activity of neuromodulators (Turchi et al., 2018), whose distributed projections affect widespread areas of the cerebral cortex. A more extended treatment of these ideas is given inLaumann & Snyder (2021).

Observations of task-related changes in FC have led to optimism about using dFC methods to decode cognitive content in the task-free state. However, we believe that caution is appropriate: changes in FC related to cognitive content in the task-free state are unlikely to be greater than task-induced modulations of FC, which are quantitatively modest (Cole et al., 2014;Gratton et al., 2016,^2018^). For example, inGratton et al. (2018), we examined the similarity of FC in the Midnight Scan Club dataset, which included data from 9 individuals across 10 days who completed 5 different tasks on each day. Task performance was associated with significant changes in FC, but these were substantially smaller than stable differences across individuals. Common task modulations and day-to-day variability each explained ~5% of the total variance in FC across session and subject. Individual participants showed idiosyncratic modulations across task states that were larger than the common effects, but these effects explained only ~10% of total variance. Each of these factors was dwarfed by stable differences across people, which accounted for 50% or more of the variance. Similarly,Cole et al. (2014)found that a stable core of FC architecture accounted for >80% of the variance in group-average estimates of FC across task and rest states in Human Connectome Project data. These findings suggest that changes in the correlation structure of BOLD signals attributable to task state are present but relatively subtle. It is our perspective that these small effect sizes combined with the unreliability of FC estimates at short time scales (Fig. 3) indicate that large amounts of data are likely to be needed for reliable detection of dFC effects related to cognition.

### 3.2 Arousal alters functional connectivity

Interpretation of dFC must also consider the effects of changes in arousal. Graded levels of arousal are reflected as changes in FC to varying degrees. Relatively large changes in FC are associated with coma or other disorders of consciousness (Boly et al., 2009;Martinez et al., 2020;Pizoli et al., 2011) and drug-induced sedation (Boveroux et al., 2010;Mhuircheartaigh et al., 2010;Palanca et al., 2015). Transitions in sleep stage are associated with moderate changes in FC (Larson-Prior et al., 2009;Samann et al., 2011). In particular, slow wave sleep (SWS) leads to increased FC in primary cortical areas more than in higher order areas (Boly et al., 2012;Tagliazucchi & Laufs, 2014). This focality is especially clear in measures of BOLD fluctuation amplitude (Fukunaga et al., 2006;Tsai et al., 2014). Arousal-dependent changes in FC are sufficiently robust that it is possible to sleep stage on the basis of fMRI (Tagliazucchi & Laufs, 2014). Using this classifier, it has been demonstrated that one third of nominally awake subjects enter stage I sleep within 3 min of resting-state scan onset if the eyes are closed.

Significant, but more modest, changes in FC are associated with arousal fluctuations during wake (e.g., associated with caffeine intake, drowsiness) (Poldrack et al., 2015;Rack-Gomer et al., 2009), opening versus closing of the eyes (Bianciardi et al., 2009;Laumann et al., 2015), and diurnal rhythms (Shannon et al., 2013). Of note, relatively higher within-subject resting-state FC variability has been found to localize to primary motor and visual areas (Hindriks et al., 2016;Laumann et al., 2015), consistent with these arousal patterns. Given these findings, we believe that arousal should be considered as a common source of neurally driven non-stationarity in FC.

In contrast to the relatively slow changes in FC described above, which vary on the order of minutes to hours, large-scale first-order BOLD signal patterns have also been used to track transient arousal events (Chang et al., 2016). Using simultaneous EEG-fMRI, it has been shown that global BOLD signal transients are associated with discrete electrophysiological signatures linked to neuromodulatory activity (Liu et al., 2018). Thus, large and rapid first-order changes in BOLD fMRI may relate to arousal. Importantly, however, levels of arousal modulate respiration, heart rate, and head motion. The global BOLD signal is also substantially affected by these same physiological measures (Birn et al., 2006;Power et al., 2017;Wise et al., 2004). Thus, arousal, cardiopulmonary physiology, movement, and BOLD signals are fundamentally entangled (Power et al., 2018;Raut et al., 2021), which complicates interpretation of observed dFC effects. For example, does arousal-related neural activity directly drive global FC dynamics, or are BOLD fMRI dynamics indirectly affected by changes in respiration? Investigation into such questions will be essential to the future of the field (seeJoliot et al. (2024)for one such recent exploration).

Despite the abundant evidence that arousal levels influence FC, arousal is still largely ignored in studies of FC dynamics. In our survey of the recent literature (Fig. 2), only 8% of studies monitored arousal during resting-state scans. Further, 46% collected rest in the eyes closed state, which is known to hasten descent into sleep (Tagliazucchi & Laufs, 2014), while 29% did not specify eye state. Arousal was considered as a potential cause for dFC differences in only 8% of studies. Given that arousal is a likely cause of non-stationarity in FC (e.g., as measured using kurtosis;Laumann et al., 2017), we suggest that arousal should be systematically monitored and considered in the interpretation of dFC in future work.

## 4 Interpretation of DFC

Given the evidence discussed in Sections2and3, we now consider applications of dFC. We consider two primary applications represented in the literature: (1) using dFC to track spontaneous cognition and (2) using dFC as a biomarker in neurological and psychiatric disorders.

### 4.1 Interpretation of dFC in relation to unconstrained cognition

A robust literature in cognitive neuroscience links electrophysiology and task-based fMRI with distinct cognitive processes (Beppi et al., 2021;Huettel, 2012). Thus, it is natural to extend this agenda to correlations in BOLD signals observed at rest, that is, resting-state functional connectivity. The notion that spontaneous FC dynamics track the content of consciousness has been articulated more than once (Fazelpour & Thompson, 2015;Northoff et al., 2020;Rabinovich et al., 2015).

Given these considerations, we briefly discuss two primary lines of evidence that have connected spontaneous BOLD signals and ongoing cognition. One line of evidence linking resting-state fMRI signals and cognition relies on associations between fMRI signals and spontaneous fluctuations in electrophysiology or task performance (Falahpour et al., 2018;Goodale et al., 2021;Keilholz, 2014;Kucyi et al., 2017;Sadaghiani et al., 2010). For example, a recent study of “brain decoding” has demonstrated that resting-state fMRI signals are modestly predictive of cognitive content (self-relevance and valence) (Kim et al., 2024). These studies provide evidence that it is possible to find associations between fMRI signals and unconstrained cognition.

Importantly, however, in the above cited experiments, the fMRI measure of interest is a map of brain activity, that is, a first-order measure of BOLD signals, not a measure of FC (seeBox 2). The only difference between such experiments and conventional task-based fMRI is that changes in cognitive state are spontaneously generated within the subject rather than experimentally imposed. Thus, these experiments demonstrate correlations between ongoing cognitive content and*first-order*measures of resting-state BOLD signals. However, they do not demonstrate a link between*dFC*(i.e., fluctuations in a second-order statistic) and cognition.

A second line of research explicitly studies the association between changes in FC and “mind-wandering” (e.g.,Gonzalez-Castillo et al., 2021;Kucyi, 2018). In many experiments, post-scan debriefing^†^is used to assess the content of thought during a resting-state scan. In these experiments, a modest correspondence between static FC measures and reported thought content has been found (Gorgolewski et al., 2014;Wang et al., 2018) (highest canonical correlation r = 0.28 in Wang et al.). Another dataset of this type has been acquired (Cremona et al., 2023;Tsuchida et al., 2021), but results relating cognitive content to changes in FC from these data have not yet been published. These studies may provide a link between spontaneous cognition and resting-state FC. However, these experiments are cross-sectional as they examine relations between FC and debriefing responses across people. It is, therefore, unclear whether such relations are driven by state differences in FC within individuals (e.g., a sad memory a person was thinking about during the preceding scan) or from trait differences in FC across individuals (e.g., propensity for negative thinking relating to that person’s intrinsic network architecture;Lynch et al., 2024). This complicates the interpretation of observed effects, given that trait effects on FC are known to be large (Finn et al., 2015;Gratton et al., 2018;Miranda-Dominguez et al., 2014;Seitzman et al., 2019).

Thus, while it may be intuitively appealing to use dFC to track spontaneous cognition, as has been done with first-order BOLD signals, we believe that additional evidence is needed to establish a strong link between these measures. In evaluating these findings, it is important to remember that, while all cognition arises from neural activity, not all neural activity necessarily relates to cognition. Moreover, FC (a second-order statistic) represents only one particular measure of neural activity, the physiological correlates of which remain an ongoing matter of debate (Pezzulo et al., 2021). We have previously argued that spontaneous neural activity (on which resting-state FC depends) is more closely associated with mechanisms related to plasticity than ongoing conscious content (Laumann & Snyder, 2021). Thus, in our view, FC (or dFC) need not be primarily related to ongoing spontaneous cognition.

We believe that a crucial challenge in relating dFC to spontaneous ongoing cognition is**time scale**. Cognitive processes evolve on a time scale of 10s–100s of milliseconds (Hammer et al., 2024;Snyder et al., 1995). As such, first-order BOLD responses, which can be measured on a time scale of several seconds, or approaches with faster temporal resolutions (e.g., EEG, ECoG) are well suited to track cognitive content, albeit typically requiring event averaging to demonstrate robust effects. However, fMRI dFC may be better suited to measuring brain processes that evolve on a time scale of minutes or more, such as variations in arousal, mood, or in broad cognitive modes. Use of dFC to track spontaneous cognitive processes must address both methodological limitations (e.g., small signal changes associated with cognitive effects on FC, mismatch between the time scale of cognition and the time required to reliably estimate FC) and interpretative ambiguities (e.g., determining whether effects are driven by states or traits, and whether they relate to arousal or other phenomena, rather than specific cognitive content). As we will discuss inSection 5, extensive repeated measures designs may be one strategy to address these concerns.

### 4.2 On the use of dFC as a biomarker

The predominant application of dFC is as a biomarker of neurologic or psychiatric disorders (for review seeFilippi et al., 2019); in our literature survey, 81% of studies measured FC dynamics in clinical populations, usually in comparison with controls. For example, it has been hypothesized that excessive rumination in the setting of depression may manifest as abnormal FC dynamics (Kaiser et al., 2016). Thus, there might be differences in the rate of measured FC changes or dwell time of different FC states. It has been reported that adding information about dynamics of FC to static measures may aid in prediction of behavioral variables (Ikeda et al., 2022;Pervaiz et al., 2022;Vidaurre et al., 2021).

However, treating dFC as a potential biomarker must contend with the statistical hazards raised inSection 2.1and the problem of artifact discussed inSection 2.2. As recently noted, static FC associations with many cognitive and neuropsychiatric traits are small (Marek et al., 2022) and limited by the reliability of FC measures themselves (Choe et al., 2017;Gell et al., 2023;Honari et al., 2019), although reliability may be improved by multivariate strategies (Spisak et al., 2023). Thus, the extent to which dFC measures will be more replicable and informative than static FC regarding trait characteristics is uncertain.

There is also an important interpretative consideration regarding the association of FC dynamics with psychiatric or behavioral variables. As discussed above, fluctuations in spontaneous fMRI signals are intrinsically entangled with physiological variables, for example, drowsiness and respiratory pattern, which themselves may be closely coupled with fMRI variables of interest (Power et al., 2018). Altered sleep dynamics are common in many neuropsychiatric syndromes. For example, individuals with Parkinson’s disease are known to have excessive daytime sleepiness and rapid-onset REM in sleep latency testing (da Silva-Junior et al., 2014). Similarly, sleep disturbance is often a core feature of depression. Further, respiratory patterns may be affected by various behavioral states, for example, anxiety. Therefore, an observation of correlation between dFC measures and a psychiatric or behavioral variable may represent a secondary effect of arousal or fluctuating arterial pCO_2_on BOLD signals (Birn et al., 2006;Wise et al., 2004) and not necessarily a primary manifestation of neuropathology. If non-specific physiological variables are driving dFC, then biomarkers premised on dFC will have limited ability to predict treatment targets or provide an understanding regarding underlying neuropathology. Future studies should consider these potential alternative explanations of dFC associations to improve biomarker development.

## 5 Conclusion and Future Directions

We have discussed issues arising in the measurement and interpretation of BOLD dFC. Functional connectivity demonstrably changes on the scale of minutes, with fluctuations in arousal and task state, albeit to a modest degree. Sampling error is a significant problem in the measurement of even static FC, which is further exacerbated in sliding window dFC (a fourth-order statistic). Moreover, fMRI data are variably contaminated with artifacts that can, if not adequately suppressed, lead to the false appearance of FC dynamics.

We believe that applications of dFC must contend with these measurement issues. Given the lower levels of reliability associated with dFC measurements, identification of dFC biomarkers likely requires substantially larger effect sizes than static FC. Use of dFC to track spontaneous cognition must further contend with interpretative issues regarding whether dFC effects reflect participant traits, specific cognitive content, broad modes of cognition, or alterations in arousal.

To make these points concrete, one might consider an experimental observation of significant differences in dFC between depressed and non-depressed individuals. At first blush, the results may appear intuitively interpretable, connecting with prior psychological theories on the role of rumination in depression. However, the present considerations raise the possibility of alternative interpretations. For example, one might ask, (1) are the results associated with differences in static FC and/or has an appropriate null model been used in the analysis? A parsimonious explanation of observed effects might be that differences in static functional network organization distinguish the two groups. (2) Were sufficient data collected and denoising performed to rule out sampling variability and artifacts? If not, it is reasonable to question whether non-neural effects drive differences between the two groups, such as differences in respiration rates or movement in the scanner. (3) Could observed differences between the two groups be explained by non-cognitive factors (e.g., sleepiness in the scanner)? Given that arousal alters FC, often to a greater degree than task state, such factors are likely to be at least as important as psychologically motivated constructs. Thus, a strong interpretation of observed results would require, at the least, additional analyses to exclude these alternative explanations.

In view of the preceding considerations, we suggest that future studies of BOLD fMRI dynamics would benefit from adopting a similar line of questioning. Particularly promising avenues for addressing these issues include experimental designs incorporating extended data collection to overcome sampling variability (Laumann et al., 2015), effective denoising methods to reduce non-neural artifacts (Parkes et al., 2018;Power et al., 2014;Satterthwaite et al., 2019), and appropriate null models to test statistical significance (Ladwig et al., 2022;Laumann et al., 2017;Liegeois et al., 2017). Moreover, most prior dFC studies have been cross-sectional in design. Designs with repeated measurements within individuals may be particularly useful to address the interpretative complexities associated with dFC. For example, detecting a dFC biomarker of depression will likely be more successful if multiple measurements are made within a person in different mood states. Similarly, identifying FC markers of spontaneous cognition would benefit from repeated sampling within individuals to determine whether differences are driven by states rather than traits. Beyond design considerations, we advocate continued exploration of the effect of arousal on BOLD dynamics both to potentially address this phenomenon as a confound in relation to FC dynamics and as an important neurally driven process itself worthy of investigation (Chang et al., 2016;Coppola et al., 2022;Joliot et al., 2024;Tarun et al., 2021).

We also recognize the importance of continued work developing novel methods for tracking BOLD dynamics that are not dependent on sliding window analyses. Some of these methods (Box 1), for example, autoregressive modeling of lag structure (Liegeois et al., 2019) and HMM (Vidaurre et al., 2017) avoid the inflated sampling error concern intrinsic to sliding window dFC (seeFigs. 3and4). Combining these methods with multivariate analysis strategies may prove additionally useful in increasing the reliability of associations between functional connectivity dynamics and behavioral measures, as has been true for other FC-based approaches (Spisak et al., 2023, but seeTervo-Clemmens et al., 2023). In our view, incorporation of null models and consideration of arousal in the interpretation of observed effects are likely to be important regardless of the approach.

## Supplementary Material

Supplementary Material

## Data Availability

Code used for generating analyses presented in this manuscript can be obtained upon reasonable request from the corresponding author.

## Footnotes

* Intrinsic time scales vary over the brain with faster activity represented in primary sensorimotor areas and slower activity represented in “higher order” regions. This difference in intrinsic time scales affects the quantity of data required to achieve a given level of FC reliability attributable to sampling error (Raut et al., 2020;Van De Ville et al., 2021).

† Establishing a one-to-one temporal correspondence between cognitive content and BOLD FC during the resting state is challenging because of basic constraints in experimental design. On the one hand, contemporaneous experience sampling during BOLD FC measurement disrupts the stream of consciousness. On the other hand, post-scan debriefing is temporally offset from the FC measure.
