# Supplementary material for: Challenges in the measurement and interpretation of dynamic functional connectivity
Source: Imaging Neurosci (Camb). 2024 Nov 19;2:imag-2-00366. doi: 10.1162/imag_a_00366 (PMC12315734; doi:10.1162/imag_a_00366)
Supplement: Supplementary Material [file imag_a_00366-supp.pdf]

Title: Challenges in the measurement and interpretation of dynamic functional connectivity

Authors: Timothy O. Laumann, Abraham Z. Snyder, Caterina Gratton

Supplemental Methods

Survey of dFC articles published in 2022 and 2023

We conducted a limited survey of recent dynamic functional connectivity studies published in 2022 and the first half of 2023 to assess the current state of the dynamic functional connectivity literature. To this end, we conducted a PubMed query on July 7, 2023 using the following search terms: ("dynamic functional connectivity") AND fMRI AND (("2023"[Date - Publication] : "3000"[Date - Publication])). We then expanded this literature review to include a broader range of time, through a PubMed query on December 18, 2023 with the same terms, but restricted to "2022"[Date-Publication].

With these search terms, we Identified 181 papers in 2022 and 85 articles in the first half of 2023, for a total of 267 articles in our review. Each article was reviewed by the three authors; the first 80 articles were reviewed by two separate raters each and discussed to assure consensus in rating decisions. We excluded (1) reviews or editorials, (2) articles that did not investigate resting-state dFC as defined in Box 1 of the manuscript, (3) articles that were not based on BOLD fMRI data from human participants (including simulation/modeling studies), and (4) articles not written in English, (5) repeated articles across searches. Based on these criteria, 63 articles were excluded (Reviews/editorials, N = 14; not resting-state, N = 18; not BOLD fMRI in humans, N = 18; not in English, N = 1; repeated across searches N = 12). The remaining 204 articles are listed in Supp. Table 1.

For each article, we determined aspects of the general design and analysis, denoising strategies, and issues regarding arousal differences in data collection. Design information included (1) the dFC method employed by the paper (e.g., sliding window, CAPs, HMM), (2) the window size used (if applicable), (3) the population studied, (4) the total sample size of the study, and (5) the amount of fMRI data collected from each participant. For denoising, we evaluated (1) what variables were included for nuisance regression (e.g., white matter, global signal, etc.), (2) if a motion censoring strategy was employed, (3) if subject exclusion was employed (and if so, whether it was based on excessive micro-movements or total size of macro-movements). For arousal, we evaluated (1) whether data was collected with eyes closed or open, (2) if arousal was monitored during data collection, and (3) if arousal was considered in the interpretation (e.g., discussion included consideration of "arousal", "sleep", or "wake" as potentially affecting study results). Note that when multiple datasets with different parameters were included in the same manuscript, these were tallied separately, unless a very large number was included (> 3), in which case these were tallied as "variable by dataset". When experimental methods were not made explicit in a manuscript, the authors examined relevant prior references to identify information to the extent possible; if still unclear, these were listed as "not specified".

Supplemental Table 1: A list of manuscripts included in our survey of dynamic functional connectivity articles from 2022 and the beginning of 2023 (see *Supp. Methods* for a specific description of search and inclusion criteria). In total, 204 manuscripts were included.

PMID	Authors	Journal	Publication Year
35069176	Xing C, Chen YC, Shang S, Xu JJ, Chen H, Yin X, Wu Y, Zheng JX.	Front Aging Neurosci	2022
34997915	Yang W, Xu X, Wang C, Cheng Y, Li Y, Xu S, Li J.	Brain Imaging Behav	2022
34051395	Marchitelli R, Paillère-Martinot ML, Bourvis N, Guerin-Langlois C, Kipman A, Trichard C, Douniol M, Stordeur C, Galinowski A, Filippi I, Bertschy G, Weibel S, Granger B, Limosin F, Cohen D, Martinot JL, Artiges E.	Biol Psychiatry Cogn Neurosci Neuroimaging	2022
34962021	Luo Y, Guo Y, Zhong L, Liu Y, Dang C, Wang Y, Zeng J, Zhang W, Peng K, Liu G.	Eur J Neurol	2022
36057586	Wang Q, Chen B, Zhong X, Hou L, Zhang M, Yang M, Wu Z, Chen X, Mai N, Zhou H, Lin G, Zhang S, Ning Y.	Alzheimers Res Ther	2022
35398505	Zhao L, Bo Q, Zhang Z, Chen Z, Wang Y, Zhang D, Li T, Yang N, Zhou Y, Wang C.	Neuroscience	2022
35126048	Pang X, Liang X, Zhao J, Wu P, Li X, Wei W, Nie L, Chang W, Lv Z, Zheng J.	Front Neurosci	2022
35551991	Spencer APC, Goodfellow M.	Neuroimage	2022
34753176	Wang P, Wang J, Michael A, Wang Z, Klugah-Brown B, Meng C, Biswal BB.	Cereb Cortex	2022
35066092	Li YT, Chen JW, Yan LF, Hu B, Chen TQ, Chen ZH, Sun JT, Shang YX, Lu LJ, Cui GB, Wang W.	Neurosci Lett	2022
35428850	Pei Y, Peng J, Zhang Y, Huang M, Zhou F.	Sci Rep	2022
36178032	Zhang Q, Zhang W, Zhang P, Zhao Z, Yang L, Zheng F, Zhang L, Huang G, Zhang J, Zheng W, Ma R, Yao Z, Hu B.	Int J Neurosci	2022
35128568	Huang J, Cheng R, Liu X, Chen L, Luo T.	Neuroradiology	2022
35712452	Fateh AA, Huang W, Mo T, Wang X, Luo Y, Yang B, Smahi A, Fang D, Zhang L, Meng X, Zeng H.	Front Neurosci	2022
34822920	Zhang H, Tao Y, Xu H, Zou S, Deng F, Huang L, Zhang H, Wang X, Tang X, Dong Z, Wang Y, Fu X, Yin L.	J Affect Disord	2022
35615457	Zhou B, Chen Y, Zheng R, Jiang Y, Li S, Wei Y, Zhang M, Gao X, Wen B, Han S, Cheng J.	Front Psychiatry	2022
35516809	Miller RL, Vergara VM, Pearlson GD, Calhoun VD.	Front Neurosci	2022
36451372	Luo Q, Chen J, Li Y, Wu Z, Lin X, Yao J, Yu H, Wu H, Peng H.	Neuroimage Clin	2022
35937891	Li Y, Wang J, Wang X, Chen Q, Qin B, Chen J.	Front Neurosci	2022
35958666	Niu H, Li W, Wang G, Hu Q, Hao R, Li T, Zhang F, Cheng T.	Front Psychiatry	2022

35082270	Liddell BJ, Das P, Malhi GS, Felmingham KL, Outhred T, Cheung J, Den M, Nickerson A, Askovic M, Aroche J, Coello M, Bryant RA.	Transl Psychiatry	2022
35606867	Dautricourt S, Gonneaud J, Landeau B, Calhoun VD, de Flores R, Poisnel G, Bougacha S, Ourry V, Touron E, Kuhn E, Demintz-King H, Marchant NL, Vivien D, de la Sayette V, Lutz A, Chételat G; Medit-Ageing Research Group.	Alzheimers Res Ther	2022
35429738	Chen P, Chen G, Zhong S, Chen F, Ye T, Gong J, Tang G, Pan Y, Luo Z, Qi Z, Huang L, Wang Y.	J Psychiatr Res	2022
36159925	Xue K, Chen J, Wei Y, Chen Y, Han S, Wang C, Zhang Y, Song X, Cheng J.	Front Psychiatry	2022
32648539	Chen G, Chen P, Gong J, Jia Y, Zhong S, Chen F, Wang J, Luo Z, Qi Z, Huang L, Wang Y.	Psychol Med	2022
35165343	Teng J, Massar SAA, Lim J.	Sci Rep	2022
32677599	Wang YM, Cai XL, Zhang RT, Zhang YJ, Zhou HY, Wang Y, Wang Y, Huang J, Wang YY, Cheung EFC, Chan RCK.	Psychol Med	2022
34715198	Tang Q, Cui Q, Chen Y, Deng J, Sheng W, Yang Y, Lu F, Zeng Y, Jiang K, Chen H.	J Affect Disord	2022
35839637	Wei L, Zhang Y, Wang J, Xu L, Yang K, Lv X, Zhu Z, Gong Q, Hu W, Li X, Qian M, Shen Y, Chen W.	Psychiatry Res	2022
35710036	Gao Y, Sun J, Cheng L, Yang Q, Li J, Hao Z, Zhan L, Shi Y, Li M, Jia X, Li H.	J Affect Disord	2022
36061606	Pan C, Yu H, Fei X, Zheng X, Yu R.	Front Neurosci	2022
35524680	Sun J, Zhao R, He Z, Chang M, Wang F, Wei W, Zhang X, Zhu Y, Xi Y, Yang X, Qin W.	Hum Brain Mapp	2022
35669490	Li X, Yan R, Yue Z, Zhang M, Ren J, Wu B.	Front Neurosci	2022
35393711	Zheng R, Chen Y, Jiang Y, Zhou B, Li S, Wei Y, Wang C, Han S, Zhang Y, Cheng J.	J Neurosci Res	2022
36340758	Liu D, Liu X, Long Y, Xiang Z, Wu Z, Liu Z, Bian D, Tang S.	Front Neurosci	2022
35944422	Yang T, Zhang Y, Zhang T, Zhou H, Yang M, Ren J, Li L, Lei D, Gong Q, Zhou D.	Seizure	2022
35142691	Zhao C, Huang WJ, Feng F, Zhou B, Yao HX, Guo YE, Wang P, Wang LN, Shu N, Zhang X.	Neural Regen Res	2022
35463932	Jiang Y, Chen Y, Zheng R, Zhou B, Wei Y, Gao A, Wei Y, Li S, Guo J, Han S, Zhang Y, Cheng J.	Front Hum Neurosci	2022
34610370	Liu Y, Ren X, Zeng M, Li J, Zhao X, Zhang X, Yang J.	Behav Brain Res	2022
35586480	Wang J, Wang K, Liu T, Wang L, Suo D, Xie Y, Funahashi S, Wu J, Pei G.	Front Comput Neurosci	2022
34164777	Nie L, Jiang Y, Lv Z, Pang X, Liang X, Chang W, Li J, Zheng J.	Cerebellum	2022
34958033	Wang J, Wang P, Jiang Y, Wang Z, Zhang H, Li H, Biswal BB.	J Alzheimers Dis	2022

34870361	Zhang X, Liu J, Yang Y, Zhao S, Guo L, Han J, Hu X.	Hum Brain Mapp	2022
35193144	Lam YS, Li J, Ke Y, Yung WH.	Cereb Cortex	2022
36087902	Xiao J, Uddin LQ, Meng Y, Li L, Gao L, Shan X, Huang X, Liao W, Chen H, Duan X.	Neuroimage	2022
36155235	Zhong S, Chen P, Lai S, Chen G, Zhang Y, Lv S, He J, Tang G, Pan Y, Wang Y, Jia Y.	J Affect Disord	2022
35738202	Li Y, Qin B, Chen Q, Chen J.	Epilepsy Res	2022
34923200	Zhang R, Tam STS, Wong NML, Wu J, Tao J, Chen L, Lin K, Lee TMC.	Neuroimage Clin	2022
35035474	Wei HL, Tian T, Zhou GP, Wang JJ, Guo X, Chen YC, Yu YS, Yin X, Li J, Zhang H.	Neural Plast	2022
35928017	Li W, Wang C, Lan X, Fu L, Zhang F, Ye Y, Liu H, Wu K, Lao G, Chen J, Li G, Zhou Y, Ning Y.	Front Neurosci	2022
35153654	Wei Y, Zhang W, Li Y, Liu X, Zha B, Hu S, Wang Y, Wang X, Yu X, Yang J, Qiu B.	Front Neurosci	2022
35611547	Wei Y, Han S, Chen J, Wang C, Wang W, Li H, Song X, Xue K, Zhang Y, Cheng J.	Hum Brain Mapp	2022
35873806	Lin K, Jie B, Dong P, Ding X, Bian W, Liu M.	Front Neurosci	2022
35147522	Yang F, Jiang X, Yue F, Wang L, Boecker H, Han Y, Jiang J.	J Neural Eng	2022
34967488	Li H, Ding F, Chen C, Huang P, Xu J, Chen Z, Wang S, Zhang M.	Hum Brain Mapp	2022
36356823	Mahmood U, Fu Z, Ghosh S, Calhoun V, Plis S.	Neuroimage	2022
37786659	Long Z, Liu X, Niu Y, Shang H, Lu H, Zhang J, Yao L.	Cogn Neurodyn	2023
34953921	Zhao L, Xue SW, Sun YK, Lan Z, Zhang Z, Xue Y, Wang X, Jin Y.	J Affect Disord	2022
36309537	You W, Luo L, Yao L, Zhao Y, Li Q, Wang Y, Wang Y, Zhang Q, Long F, Sweeney JA, Gong Q, Li F.	Schizophrenia (Heidelb)	2022
35903058	Xue SW, Kuai C, Xiao Y, Zhao L, Lan Z.	Psychiatry Investig	2022
34929585	Ghanbari M, Soussia M, Jiang W, Wei D, Yap PT, Shen D, Zhang H.	Neuroimage Clin	2022
34700242	Anastasiou A, Cribben I, Fryzlewicz P.	Med Image Anal	2022
36367793	Chen J, Chen WY, Huang X.	Neuroreport	2022
36389084	Li H, Li L, Li K, Li P, Xie W, Zeng Y, Kong L, Long T, Huang L, Liu X, Shu Y, Zeng L, Peng D.	Front Aging Neurosci	2022
36040940	Huang H, Liu Q, Jiang Y, Yang Q, Zhu X, Li Y.	IEEE Trans Neural Syst Rehabil Eng	2022
35365757	Deng S, Li J, Thomas Yeo BT, Gu S.	Commun Biol	2022
36335047	Hao Z, Li H, Lin Y.	Psychiatry Res Neuroimaging	2022
36117618	Di Nardo F, Manara R, Canna A, Trojsi F, Velletrani G, Sinisi AA, Cirillo M, Tedeschi G, Esposito F.	Front Neurosci	2022

35169701	von Schwanenflug N, Krohn S, Heine J, Paul F, Prüss H, Finke C.	Brain Commun	2022
34314368	Ji J, Chen Z, Yang C.	IEEE J Biomed Health Inform	2022
35131700	Pervaiz U, Vidaurre D, Gohil C, Smith SM, Woolrich MW.	Med Image Anal	2022
35975454	Zhong X, Chen B, Hou L, Wang Q, Liu M, Yang M, Zhang M, Zhou H, Wu Z, Zhang S, Lin G, Ning Y.	CNS Neurosci Ther	2022
35836122	Du K, Chen P, Zhao K, Qu Y, Kang X, Liu Y; Multi-center Alzheimer Disease Imaging Consortium.	BMC Bioinformatics	2022
34303848	Fu Z, Sui J, Espinoza R, Narr K, Qi S, Sendi MSE, Abbott CC, Calhoun VD.	Biol Psychiatry Cogn Neurosci Neuroimaging	2022
35716842	Basile GA, Bertino S, Nozais V, Bramanti A, Ciurleo R, Anastasi GP, Milardi D, Cacciola A.	Neuroimage	2022
34896588	Ikeda S, Kawano K, Watanabe S, Yamashita O, Kawahara Y.	Neuroimage	2022
36118700	Xu X, Chen YC, Yin X, Zuo T, Feng G, Xu K.	Front Aging Neurosci	2022
35733435	Iraji A, Faghiri A, Fu Z, Rachakonda S, Kochunov P, Belger A, Ford JM, McEwen S, Mathalon DH, Mueller BA, Pearlson GD, Potkin SG, Preda A, Turner JA, van Erp TGM, Calhoun VD.	Netw Neurosci	2022
35845606	Ni X, Zhang J, Sun M, Wang L, Xu T, Zeng Q, Wang X, Wang Z, Liao H, Hu Y, Gao Q, Zhao L.	Front Mol Neurosci	2022
35331869	Coppola P, Spindler LRB, Luppi AI, Adapa R, Naci L, Allanson J, Finoia P, Williams GB, Pickard JD, Owen AM, Menon DK, Stamatakis EA.	Neuroimage	2022
34902547	Varangis E, Qi W, Stern Y, Lee S.	Neuroimage	2022
35759586	Zhu Q, Xu R, Wang R, Xu X, Zhang Z, Zhang D.	IEEE Trans Med Imaging	2022
35174863	Cheng B, Wang X, Roberts N, Zhou Y, Wang S, Deng P, Meng Y, Deng W, Wang J.	Cereb Cortex	2022
35504564	Jun S, Alderson TH, Altmann A, Sadaghiani S.	Neuroimage	2022
32762276	Chen B.	Int J Neurosci	2022
36324658	Suñol M, Alemany S, Bustamante M, Diez I, Contreras-Rodríguez O, Laudo B, Macià D, Martínez-Vilavella G, Martínez-Zalacaín I, Menchón JM, Pujol J, Sunyer J, Sepulcre J, Soriano-Mas C.	Biol Psychiatry Glob Open Sci	2021
36340776	Shi Y, Zeng W.	Front Neurosci	2022
36213747	Miao J, Ailes I, Krisa L, Fleming K, Middleton D, Talekar K, Natale P, Mohamed FB, Hines K, Matias CM, Alizadeh M.	Front Neurosci	2022
34838931	Liu H, Hu K, Peng Y, Tian X, Wang M, Ma B, Wu Y, Sun W, Liu B, Li A, Han R.	Behav Brain Res	2022

34752656	Aracil-Bolaños I, Martínez-Horta S, González-de-Echávarri JM, Sampedro F, Pérez-Pérez J, Horta-Barba A, Campolongo A, Izquierdo C, Gómez-Ansón B, Pagonabarraga J, Kulisevsky J.	Mov Disord	2022
35218862	Wu Y, Zheng Y, Li J, Liu Y, Liang X, Chen Y, Zhang H, Wang N, Weng X, Qiu S, Wang J.	J Affect Disord	2022
35444518	Maltbie E, Yousefi B, Zhang X, Kashyap A, Keilholz S.	Front Neural Circuits	2022
35552090	Tang J, Xia Y, Liu N, Li L, Zou P, Zhu P, Shan X, Lui S, Lu Y, Yan Z.	Psychoneuroendocrinology	2022
36845989	Jiang P, Sun J, Zhou X, Lu L, Li L, Xu J, Huang X, Li J, Gong Q.	Drug Alcohol Depend Rep	2022
35325759	Wang H, Zhu R, Tian S, Zhang S, Dai Z, Shao J, Xue L, Yao Z, Lu Q.	J Psychiatr Res	2022
34783122	Moretto M, Silvestri E, Zangrossi A, Corbetta M, Bertoldo A.	Hum Brain Mapp	2022
35329938	Hsieh H, Xu Q, Yang F, Zhang Q, Hao J, Liu G, Liu R, Yu Q, Zhang Z, Xing W, Bernhardt BC, Lu G, Zhang Z.	J Clin Med	2022
34952988	Zhang R, Chen Z, Hu B, Zhou F, Feng T.	Hum Brain Mapp	2022
35844218	Qin Y, Li S, Yao D, Luo C.	Front Neurosci	2022
35360175	Farinha M, Amado C, Morgado P, Cabral J.	Front Neurosci	2022
35705764	Luijendijk MJ, Bekele BM, Schagen SB, Douw L, de Ruiter MB.	Brain Imaging Behav	2022
36138998	Ouyang X, Long Y, Wu Z, Liu D, Liu Z, Huang X.	Brain Sci	2022
36516329	Yin Y, He S, He N, Zhang W, Luo L, Chen L, Liu T, Tian M, Xu J, Chen S, Li F.	Oral Dis	2022
34155908	Savva AD, Matsopoulos GK, Mitsis GD.	Brain Connect	2022
35069281	Zhao L, Wang D, Xue SW, Tan Z, Luo H, Wang Y, Li H, Pan C, Fu S, Hu X, Lan Z, Xiao Y, Kuai C.	Front Psychiatry	2022
36421852	Liu Y, Cao S, Du B, Zhang J, Chen C, Hu P, Tian Y, Wang K, Ji GJ, Wei Q.	Brain Sci	2022
35729463	Cai LM, Shi JY, Dong QY, Wei J, Chen HJ.	Brain Imaging Behav	2022
34859934	Ganesan S, Lv J, Zalesky A.	Hum Brain Mapp	2022
36601596	Zhang X, Shams SP, Yu H, Wang Z, Zhang Q.	Front Neurosci	2022
35219859	Ricchi I, Tarun A, Maretic HP, Frossard P, Van De Ville D.	Neuroimage	2022
35781077	Hancock F, Cabral J, Luppi AI, Rosas FE, Mediano PAM, Dipasquale O, Turkheimer FE.	Neuroimage	2022
36579785	Fu Y, Niu M, Gao Y, Dong S, Huang Y, Zhang Z, Zhuo C.	J Neural Eng	2022
34837154	Ghanbari M, Zhou Z, Hsu LM, Han Y, Sun Y, Yap PT, Zhang H, Shen D.	Neuroinformatics	2022
35082661	Tang S, Wu Z, Cao H, Chen X, Wu G, Tan W, Liu D, Yang J, Long Y, Liu Z.	Front Aging Neurosci	2022

35059721	Li Y, Zeng W, Deng J, Shi Y, Nie W, Luo S, Zhang H.	Cereb Cortex	2022
35221988	Xing J, Jia J, Wu X, Kuang L.	Front Aging Neurosci	2022
35959244	Huang H, Zhang B, Mi L, Liu M, Chang X, Luo Y, Li C, He H, Zhou J, Yang R, Li H, Jiang S, Yao D, Li Q, Duan M, Luo C.	Front Hum Neurosci	2022
35774557	Jensen KHR, McCulloch DE, Olsen AS, Bruzzone SEP, Larsen SV, Fisher PM, Frokjaer VG.	Front Neurosci	2022
34491896	Li Y, Liu J, Jiang Y, Liu Y, Lei B.	IEEE Trans Med Imaging	2022
36110425	Qiao J, Wang R, Liu H, Xu G, Wang Z.	Front Aging Neurosci	2022
36248645	Palmer WC, Park SM, Levendovszky SR.	Front Neurosci	2022
35756935	Li Z, Zhao L, Ji J, Ma B, Zhao Z, Wu M, Zheng W, Zhang Z.	Front Neurol	2022
36312008	Huang NX, Gao ZL, Lin JH, Lin YJ, Chen HJ.	Front Neurosci	2022
35522357	Cao P, Wen G, Liu X, Yang J, Zaiane OR.	Med Biol Eng Comput	2022
37859293	Talesh Jafadideh A, Mohammadzadeh Asl B.	Comput Biol Med	2022
34389436	Zhao Z, Zhang Y, Chen N, Li Y, Guo H, Guo M, Yao Z, Hu B.	Prog Neuropsychopharmacol Biol Psychiatry	2022
36186871	Jia H, Wu X, Wu Z, Wang E.	Front Psychiatry	2022
35938250	Li C, Li Y, Wu J, Wu M, Peng F, Chao Q; Alzheimer’s Disease Neuroimaging Initiative.	J Alzheimers Dis	2022
37786665	Zhao G, Zhan Y, Zha J, Cao Y, Zhou F, He L.	Cogn Neurodyn	2023
35405342	Pirondini E, Kinany N, Sueur CL, Griffis JC, Shulman GL, Corbetta M, Van De Ville D.	Neuroimage	2022
35250441	Li Y, Zeng W, Shi Y, Deng J, Nie W, Luo S, Yang J.	Front Neurosci	2022
35105657	Good T, Schirner M, Shen K, Ritter P, Mukherjee P, Levine B, McIntosh AR.	eNeuro	2022
35462573	Bai L, Zhang L, Chen Y, Li Y, Ma D, Li W, Meng Y, Zhao Y, Wang Y, Zeng Q, Zhuang Q.	Neuroradiology	2022
34870570	Shunkai L, Su T, Zhong S, Chen G, Zhang Y, Zhao H, Chen P, Tang G, Qi Z, He J, Zhu Y, Lv S, Song Z, Miao H, Hu Y, Jia Y, Wang Y.	Psychol Med	2023
36826627	Dai Y, Zhou Z, Chen F, Zhang L, Ke J, Qi R, Lu G, Zhong Y.	Brain Imaging Behav	2023
36162641	Zhu Z, Wang H, Bi H, Lv J, Zhang X, Wang S, Zou L.	Behav Brain Res	2023
37025377	Zhang Y, Cai X, Duan M, He H.	Front Neurosci	2023
36967436	Chen F, Chen Q, Zhu Y, Long C, Lu J, Jiang Y, Zhang X, Zhang B.	Transl Stroke Res	2023
37250690	Shi Y, Shen Z, Zeng W, Luo S, Zhou L, Wang N.	Front Hum Neurosci	2023
37008221	Cao Y, Si Q, Tong R, Zhang X, Li C, Mao S.	Front Neurosci	2023

35965076	Peng L, Luo Z, Zeng LL, Hou C, Shen H, Zhou Z, Hu D.	Cereb Cortex	2023
37098479	Ding Z, Ding Z, Chen Y, Lv D, Li T, Shang T, Ma J, Zhan C, Yang X, Xiao J, Sun Z, Wang N, Guo W, Li C, Yu Z, Li P.	BMC Psychiatry	2023
36708666	Liu X, Qiu S, Wang X, Chen H, Tang Y, Qin Y.	Neuroimage Clin	2023
36581179	Kang L, Wang W, Zhang N, Yao L, Tu N, Feng H, Zong X, Bai H, Li R, Wang G, Bu L, Wang F, Liu Z.	J Affect Disord	2023
35765198	Li W, Ding S, Zhao G.	Acta Radiol	2023
36919400	Jing R, Lin X, Ding Z, Chang S, Shi L, Liu L, Wang Q, Si J, Yu M, Zhuo C, Shi J, Li P, Fan Y, Lu L.	Hum Brain Mapp	2023
36761865	Xie Y, Guan M, He Y, Wang Z, Ma Z, Fang P, Wang H.	Front Psychiatry	2023
36816121	Li DJ, Huang BL, Peng Y, Liang LY, Liu H.	Front Neurosci	2023
37234210	Zheng Y, Wu Y, Liu Y, Li D, Liang X, Chen Y, Zhang H, Guo Y, Lu R, Wang J, Qiu S.	Front Psychiatry	2023
36642500	Lu F, Chen Y, Cui Q, Guo Y, Pang Y, Luo W, Yu Y, Chen J, Gao J, Sheng W, Tang Q, Zeng Y, Jiang K, Gao Q, He Z, Chen H.	Cereb Cortex	2023
37369744	Moretto M, Silvestri E, Facchini S, Anglani M, Cecchin D, Corbetta M, Bertoldo A.	Sci Rep	2023
36736793	Lin X, Jing R, Chang S, Liu L, Wang Q, Zhuo C, Shi J, Fan Y, Lu L, Li P.	J Affect Disord	2023
37350441	Bruyn N, Bonkhoff AK, Saenen L, Thijs L, Essers B, Alaerts K, Verheyden G.	Neurorehabil Neural Repair	2023
36367193	Hussain S, Langley J, Seitz AR, Hu XP, Peters MAK.	Brain Connect	2023
37021138	Wu K, Jelfs B, Mahmoud SS, Neville K, Fang JQ.	Front Neurosci	2023
36988434	Jing R, Chen P, Wei Y, Si J, Zhou Y, Wang D, Song C, Yang H, Zhang Z, Yao H, Kang X, Fan L, Han T, Qin W, Zhou B, Jiang T, Lu J, Han Y, Zhang X, Liu B, Yu C, Wang P, Liu Y; Alzheimer's Disease Neuroimaging Initiative.	Hum Brain Mapp	2023
37017941	Bai P, Wang Y, Zhao F, Liu Q, Wang C, Liu J, Qiao Y, Ma C, Ren Y.	Med Phys	2023
36481660	Sang L, Wang L, Zhang J, Qiao L, Li P, Zhang Y, Wang Q, Li C, Qiu M.	Neurobiol Aging	2023
36087094	Seeley SH, Andrews-Hanna JR, Allen JJB, O'Connor MF.	Hum Brain Mapp	2023
36741115	Xue K, Chen J, Wei Y, Chen Y, Han S, Wang C, Zhang Y, Song X, Cheng J.	Front Psychiatry	2023
36919656	Rokham H, Falakshahi H, Fu Z, Pearlson G, Calhoun VD.	Hum Brain Mapp	2023
36760808	Li Y, Li M, Zhao K, Wang Y, Tan X, Qin C, Rao Y, Sun Z, Ge L, Cao Z, Liang Y, Qiu S.	Front Endocrinol (Lausanne)	2023
36816124	Ji Y, Huang SQ, Cheng Q, Fu WW, Zhong PP, Chen XL, Shu BL, Wei B, Huang QY, Wu XR.	Front Neurosci	2023

36637216	Long Y, Ouyang X, Yan C, Wu Z, Huang X, Pu W, Cao H, Liu Z, Palaniyappan L.	Hum Brain Mapp	2023
36942648	Duval PE, Fornari E, Décaillet M, Ledoux JB, Beaty RE, Denervaud S.	Dev Sci	2023
36580711	Xu F, Qiao C, Zhou H, Calhoun VD, Stephen JM, Wilson TW, Wang Y.	Neural Netw	2023
36510701	Wan X, Zhang P, Wang W, Wu X, Tan Q, Su X, Zhang S, Yang X, Li S, Shao H, Yue Q, Gong Q.	CNS Neurosci Ther	2023
37321986	Kim J, Andrews-Hanna JR, Eisenbarth H, Lux BK, Kim HJ, Lee E, Lindquist MA, Losin EAR, Wager TD, Woo CW.	Nat Commun	2023
36698861	Penalba-Sánchez L, Oliveira-Silva P, Sumich AL, Cifre I.	Front Aging Neurosci	2023
36577226	Byun JI, Jahng GH, Ryu CW, Park S, Lee KH, Hong SO, Jung KY, Shin WC.	Sleep Med	2023
37088249	Zhu Z, Wang S, Lee TMC, Zhang R.	J Affect Disord	2023
37115390	Yuan Y, Duan Y, Li W, Ren J, Li Z, Yang C.	Brain Topogr	2023
36214186	Liu T, Shi Z, Zhang J, Wang K, Li Y, Pei G, Wang L, Wu J, Yan T.	Hum Brain Mapp	2023
37062926	Saccaro LF, Gaviria J, Ville DV, Piguet C.	Brain Behav	2023
36764224	Xin X, Feng Y, Lou Y, Feng J, Gao X.	J Psychiatr Res	2023
36472382	Ramirez-Mahaluf JP, Tepper Á, Alliende LM, Mena C, Castañeda CP, Iruretagoyena B, Nachar R, Reyes-Madrigal F, León-Ortiz P, Mora-Durán R, Ossandon T, Gonzalez-Valderrama A, Undurraga J, de la Fuente-Sandoval C, Crossley NA.	Schizophr Bull	2023
36089839	Li Y, Yu X, Ma Y, Su J, Li Y, Zhu S, Bai T, Wei Q, Becker B, Ding Z, Wang K, Tian Y, Wang J.	Cereb Cortex	2023
36611231	Petkoski S, Ritter P, Jirsa VK.	Cereb Cortex	2023
36941217	de la Cruz F, Schumann A, Suttkus S, Helbing N, Bär KJ.	Eur J Neurosci	2023
36722495	Yang D, Li J, Ke Z, Qin R, Mao C, Huang L, Mo Y, Hu Z, Lv W, Huang Y, Zhang B, Xu Y.	Hum Brain Mapp	2023
37005068	Sakiyama K, Abe N, Fujieda Y, Tha KK, Narita H, Karino K, Kanda M, Kono M, Kato M, Atsumi T.	Cereb Cortex	2023
36896755	Ghanbari M, Li G, Hsu LM, Yap PT.	Hum Brain Mapp	2023
36952467	Hancock F, Rosas FE, McCutcheon RA, Cabral J, Dipasquale O, Turkheimer FE.	PLoS One	2023
34588010	Sheng D, Pu W, Linli Z, Tian GL, Guo S, Fei Y.	Psychol Med	2023
37151225	Lorenzini L, Ingala S, Collij LE, Wottschel V, Haller S, Blennow K, Frisoni G, Chételat G, Payoux P, Lage-Martinez P, Ewers M, Waldman A, Wardlaw J, Ritchie C, Gispert JD, Mutsaerts HJMM, Visser PJ, Scheltens P, Tijms B, Barkhof F, Wink AM.	Brain Commun	2023
36642496	Yang X, Zhou X, Xin F, Becker B, Linden D, Hernaus D.	Cereb Cortex	2023

37402747	Yang M, Liu L, Cui H, Deng C, Xiong W, Zhao G, Du S, Kosten TR, Chen H, Li Z, Zhang X.	Schizophrenia (Heidelb)	2023
36790361	Tang B, Zhang W, Liu J, Deng S, Hu N, Li S, Zhao Y, Liu N, Zeng J, Cao H, Sweeney JA, Gong Q, Gu S, Lui S.	Cereb Cortex	2023
37128187	Liu H, Zhang G, Zheng H, Tan H, Zhuang J, Li W, Wu B, Zheng W.	J Neurotrauma	2023
37232486	Ramkiran S, Veselinović T, Dammers J, Gaebler AJ, Rajkumar R, Shah NJ, Neuner I.	Hum Brain Mapp	2023
36963740	Wu H, Xie Q, Pan J, Liang Q, Lan Y, Guo Y, Han J, Xie M, Liu Y, Jiang L, Wu X, Li Y, Qin P.	Neuroimage	2023
35368068	Sastry NC, Roy D, Banerjee A.	Cereb Cortex	2023
37008205	Chen XM, Wen Y, Chen S, Jin X, Liu C, Wang W, Kong N, Ling DY, Huang Q, Chai JE, Zhao XL, Li J, Xu MS, Jiang Z, Du HG.	Front Neurosci	2023
37436866	Cao T, Lin R, Zheng Y, Shen D, Xu L.	IEEE Trans Biomed Eng	2023
36456762	Simos NJ, Manolitsi K, Luppi AI, Kagialis A, Antonakakis M, Zervakis M, Antypa D, Kavroulakis E, Maris TG, Vakis A, Stamatakis EA, Papadaki E.	Neuroinformatics	2023
37245549	Liu C, Belleau EL, Dong D, Sun X, Xiong G, Pizzagalli DA, Auerbach RP, Wang X, Yao S.	J Affect Disord	2023
36905309	Yang L, Liu G, Li S, Yao C, Zhao Z, Chen N, Zhang P, Shang Y, Wang Y, Zhang D, Tian X, Zhang J, Yao Z, Hu B.	Eur J Neurol	2023
36610729	Li X, Jia X, Liu Y, Bai G, Pan Y, Ji Q, Mo Z, Zhao W, Wei Y, Wang S, Yin B, Zhang J, Bai L.	Cereb Cortex	2023
37005067	Yuan B, Xie H, Gong F, Zhang N, Xu Y, Zhang H, Liu J, Chen L, Li C, Tan S, Lin Z, Hu X, Gu T, Cheng J, Lu J, Liu D, Wu J, Yan J.	Cereb Cortex	2023
37363818	Dirkx MF, Shine JM, Helmich RC.	Mov Disord	2023
36329223	Siffredi V, Liverani MC, Freitas LGA, Tadros D, Farouj Y, Borradori Tolsa C, Van De Ville D, Hüppi PS, Ha-Vinh Leuchter R.	Pediatr Res	2023
37334006	Asadi N, Olson IR, Obradovic Z.	Netw Neurosci	2023
36307355	Sacca V, Zhang Y, Cao J, Li H, Yan Z, Ye Y, Hou X, McDonald CM, Todorova N, Kong J, Liu B.	Neuromodulation	2023
36801417	Kyuragi Y, Oishi N, Yamasaki S, Hazama M, Miyata J, Shibata M, Fujiwara H, Fushimi Y, Murai T, Suwa T.	J Affect Disord	2023
